# Characterization of Distant and Moderate Earthquakes with Inexpensive MEMS Sensors: Application to the M_w_ 6.3, 29th December 2020, Petrinja Event

**DOI:** 10.3390/s22114166

**Published:** 2022-05-30

**Authors:** Valeria Cascone, Jacopo Boaga

**Affiliations:** 1 Department of Geosciences, University of Padova, Via G. Gradenigo 6, 35131 Padova, Italy; jacopo.boaga@unipd.it; 2 Isamgeo Italia s.r.l., Via Arno 1, 21021 Angera, Italy

**Keywords:** seismic network, seismic ground motion, MEMS sensors, PGA

## Abstract

In this work, we evaluate the suitability of a new MEMS sensor prototype, called ASX1000 (ADEL s.r.l., Modena, Italy), for the monitoring of distant and moderate seismic events. This device is an inexpensive capacitive accelerometer with a relatively low level of instrumental noise; it can record both local and far seismic events. An experimental network built with ASX1000 MEMS, located in northern Italy, was able to record the M_w_ 6.3 Petrinja earthquake that occurred in December 2020; it had an epicentral distance of more than 350 km. We retrieved the strong motion parameters (PGA, pseudo-absolute velocity, and pseudo-absolute spectral acceleration) from the acceleration time histories recorded by the MEMS sensors. The obtained parameters were compared with the ones obtained by the closer high-quality seismometers, belonging to the INGV National Seismic Network. The comparison to the highest-quality sensors confirms a reasonable agreement of the inferred parameters. This work suggests that—in the near future—MEMS sensors could be adopted to integrate the existing seismic network. A denser coverage of sensors can sample more accurately the seismic wavefield, taking into account the large spatial variability of local geology and the relative differences in seismic response.

## 1. Introduction

Earthquake engineering needs accurate measurements of seismic ground motions to achieve detailed characterizations of the local responses [1]. The mapping of the distribution of the earthquake’s effects, with adequate resolution, depends on the availability and spatial distributions of the monitoring stations [2,3,4,5]. Because of the high costs of advanced seismometers (considering the purchase, the installation, and the maintenance costs), the number of sensors generally deployed in the national seismic networks is relatively low. This implies a limited spatial sampling of the ground motion. To mitigate the high costs of installing standard seismic arrays using conventional seismometers, inexpensive sensors (e.g., micro-electro-mechanical system (MEMS) accelerometers) have recently been implemented [6]. MEMS accelerometers are devices with very small footprints (micrometers to a few millimeters in size) and with low power consumption [7]. Among the various typologies of MEMS accelerometers (such as piezoelectric, piezoresistive, and strain gauge), the electromechanical piezoresistive or capacitive sensors are the most widely adopted [8,9]. Capacitive accelerometers are based on spring mass-like systems placed on silicon substrates [10]. MEMS are inexpensive products: their costs are generally two orders of magnitude less than high-quality seismic stations. These accelerometers are extremely popular and are adopted in a variety of industrial fields (monitoring of machines and vehicles, mobile phones, game controllers, etc.) [3,4,5,6].

Several dense low-cost MEMS networks have been recently tested for the monitoring of strong earthquakes and ground shaking, with promising results [11,12,13,14,15,16,17]. However, in these works, the epicentral distances of the seismic events, concerning the sensors, do not exceed 100 km. Thus, the question remains as to whether such sensors are suitable for distant earthquake detecting.

Cascone et al. [18] presented a new MEMS sensor prototype, named ASX1000, designed and built by ADEL s.r.l. (an Italian-based company specializing in telecommunications technology). This device is an inexpensive (USD 500) capacitive accelerometer with a low level of internal instrumental noise. A total of 15 of these prototypes were installed in Italy and they recorded major earthquakes as well as small local seismic events. The most energetic seismic event recorded by the MEMS sensor array is the M_w_ 6.3 29 Petrinja earthquake that took place in December 2020. The epicenter of this seismic event is located ~350 km away from the MEMS sensor array. In this work, we present the recordings of this distant seismic event and compare the responses of the MEMS with the ones inferred by high-quality seismic stations. 

## 2. Methods

**Figure 1:**
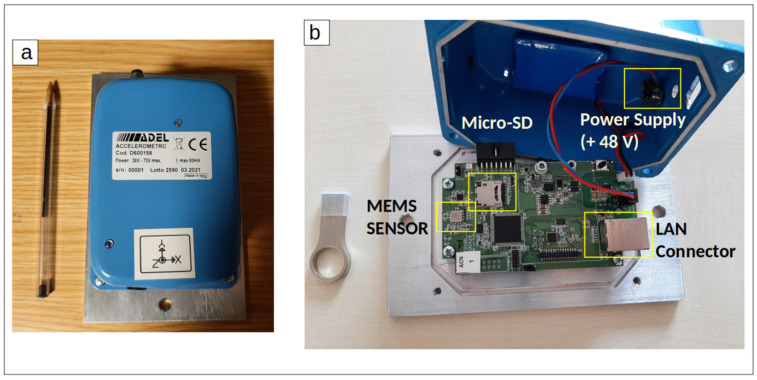
(**a**) ASX1000 MEMS sensor prototype; (**b**) its internal circuit batch.

The MEMS sensor prototype adopted in this work, named ASX1000, is a low-noise density triaxial multi-range accelerometer; it is characterized by low power consumption (Table 1). It has an internal circuit of transduction, providing digital output. The device adopted in this study is shown in Figure 1a. The circuit was mounted on a metal plate to be fixed; the cover is completely hermetic and dust/waterproof. Figure 1b shows the internal circuit batch. 

This sensor operates in a high sensitivity mode for an acceleration dynamic range of ±2 g, but it also supports the ±4 g full scale. This value guarantees that the sensor is even capable of recording very strong accelerations, such as those induced by a strong local earthquake (M > 6.5), remaining in a linear regime (Table 1). The bandwidth is (up to) 62.5 Hz (Table 1). This bandwidth falls into the scope of common earthquake engineering since the frequencies of interest for soil/building interactions are limited to narrower ranges (approximately 0.1–20 Hz). Other technical specifications, such as the internal noise and the calibration test performed on the shake table, are described in detail by Cascone et al. [18]. We summarize the technical features of the ASX1000 MEMS sensor prototype in Table 1.

**Figure 2:**
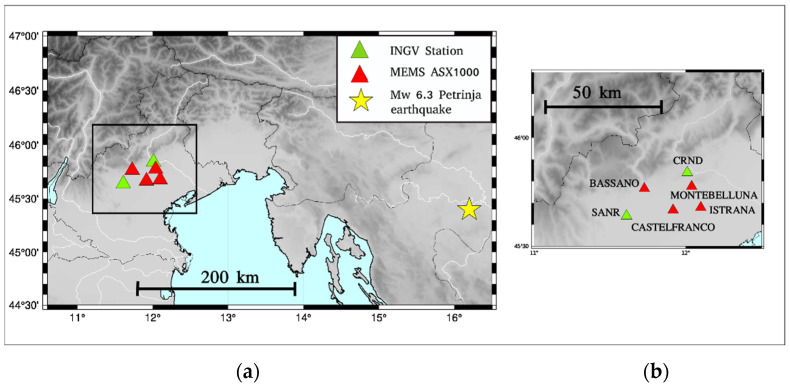
(**a**) Location of the ASX1000 prototype in northern Italy (red triangle). The green triangles are high-quality sensors belonging to INGV seismic networks, respectively. The epicenter of the M_w_ 6.3 Petrinja earthquake (December 2020) is indicated with the yellow star. (**b**) The seismic sensors located in the Veneto region, and the corresponding IDs.

**Figure 3:**
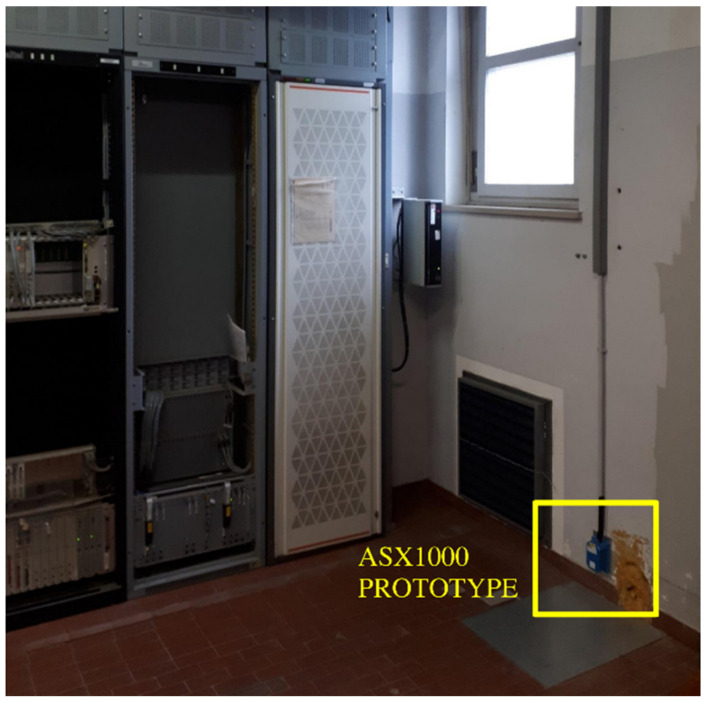
The ASX1000 prototype (yellow square) installed inside a local telecommunication infrastructure.

In January 2020, two experimental arrays of MEMS sensors were installed in two seismic active areas of Italy (a European country with high seismic risk): the inner part of the Umbria valley (Central Italy, [19]) and the eastern–southern Alpine front (northern Italy [20,21]) (Figure 2). The MEMS sensors are installed inside telecommunication infrastructures at the base of the local server room (located on the bottom floor of the building), firmly coupled to the ground with screws and plugs (Figure 3). Raw data are transmitted in real-time via a LAN connection to a central service managed by the ADEL company.

In this work, due to the location of the M_w_ 6.3 29 Petrinja earthquake (December 2020), we will only consider the northern Italy array. Figure 2 shows the locations of the seismic event, the MEMS array, and the closest national strong-motion high-quality seismometers. The high-quality broadband sensors belong to the National Seismic Network managed by the INGV (Istituto Nazionale di Geofisica e Vulcanologia, Rome, Italy, red triangles).

**Figure 4:**
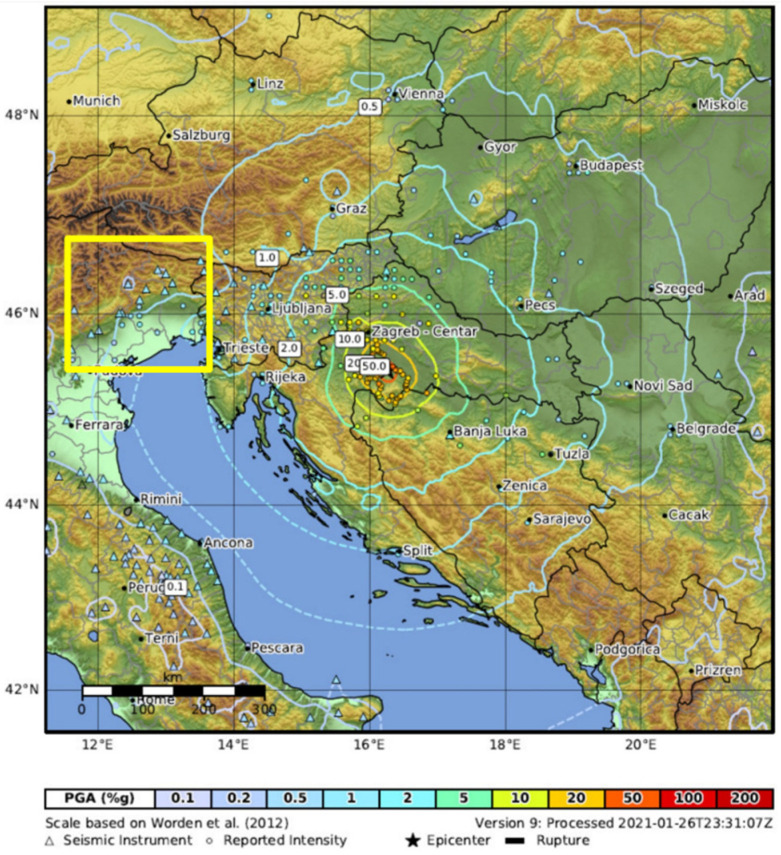
The peak ground acceleration map inferred from the USGS shake map (modified). In the yellow square, we marked the seismic station installed in northern Italy.

During the last two years, the sensors recorded more than 50 seismic events with magnitudes between 1.5 and 6.3. The highest magnitude recorded by the ASX1000 prototype (and the objective of this study) was the M_w_ 6.3 Petrinja earthquake that took place on 29 December 2020 (11:19:54 UTC). The epicenter of this seismic event is displayed in Figure 2 with a yellow star. The shake map of the M_w_ 6.3 Petrinja earthquake (December 2020), as produced by the USGS, is shown in Figure 4. The map provides the values of peak ground acceleration (PGA), highlighting the distribution and severity of the shacked area. The map includes the seismic station data belonging to the Italian Seismic Network (see Data Availability Statement).

## 3. Results

The ASX1000 prototypes array installed in northern Italy was able to record the distant and moderate seismic events of the M_w_ 6.3 Petrinja earthquake (29 December 2020).

**Figure 5:**
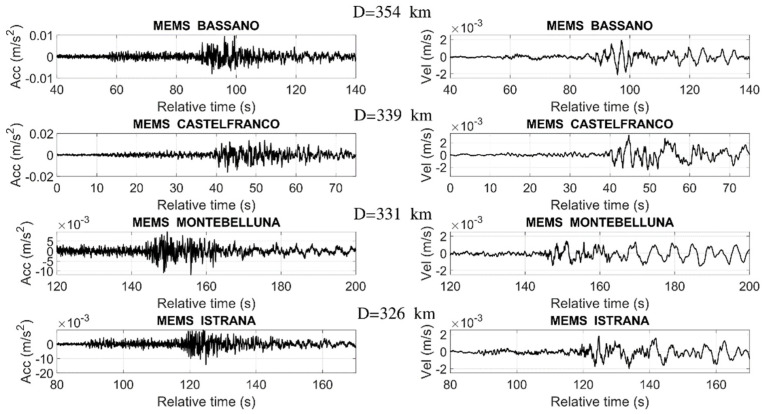
Acceleration time histories of the M_w_ 6.3 Petrinja earthquake that took place on 29 December 2020, recorded by the MEMS sensor installed in northern Italy (D indicates the epicentral distances).

In Figure 5, we plot the waveforms (acceleration and velocity time histories) of the seismic events recorded by the ASX1000 prototypes located in the Bassano, Castelfranco, Montebelluna, and Istrana stations (here, we compare the horizontal transverse components). The corresponding epicentral distances are also reported, showing that the highest epicentral distance is equal to 354 km. 

From the recorded acceleration time histories, we retrieved the main ground motion parameters: peak ground acceleration (PGA), pseudo-absolute spectral acceleration (Sa), and pseudo-absolute spectral velocity (Sv). PGA quantifies the maximum acceleration experienced by a particle on the ground. Sa and Sv represent approximately the seismic effects experienced by a building, modeled by a particle mass on a mass-less vertical rod having the same natural period of vibration as the building. PGA is easily inferred considering the absolute value of the amplitude of the acceleration time history. Sa and Sv are inferred with a Matlab [22] code based on the Newmark-beta method with linear acceleration (γ factor = 1/2, β factor = 1/6). This method allows obtaining pseudo-absolute acceleration and velocity based on the maximum displacement captured for each period (T), with damping of 5%.

A comparison with the high-quality available stations was then made. For this analysis, we considered the broadband seismic stations installed in the Veneto region (northern Italy) belonging to INGV (see Figure 2). In particular, we considered the closest available INGV stations: CRND and SANR (Kinematic Episensors instrument). For the velocimeter sensors, a unit conversion in acceleration was made.

Since soil types strongly modify the local responses [23,24,25,26], we limited the comparison to stations that had similar geological conditions. The local telecommunication infrastructures where the ASX1000 prototypes are installed are located on soil types B or C [27]. The MEMS sensor-based network of northern Italy is located in the pre-Alpine zone, where gravelly soil sediments are predominant [25]. SANR and CRND high-quality seismic stations are located on class B soils (see Data Availability Statement). For the comparison, we selected a couple of MEMS and the closest high-quality sensors with the same soil classes: CASTELFRANCO-CRND and BASSANO-SANR. In Table 2, we present the three components of PGA and PGV recorded by the ASX1000 prototypes with the ones recorded by the INGV high-quality seismic stations.

**Figure 6:**
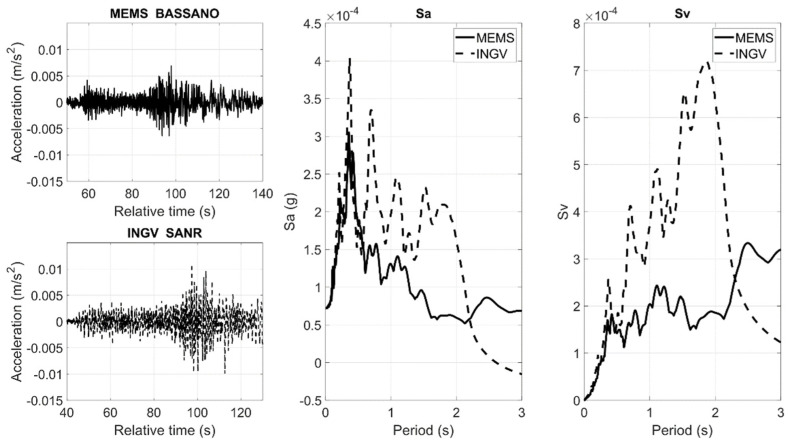
Comparison of the ASX1000 MEMS sensor prototype, BASSANO, and the high-quality sensors, SANR, belonging to the INGV networks. The comparison is made in terms of waveforms, normalized Sa, and Sv.

**Figure 7:**
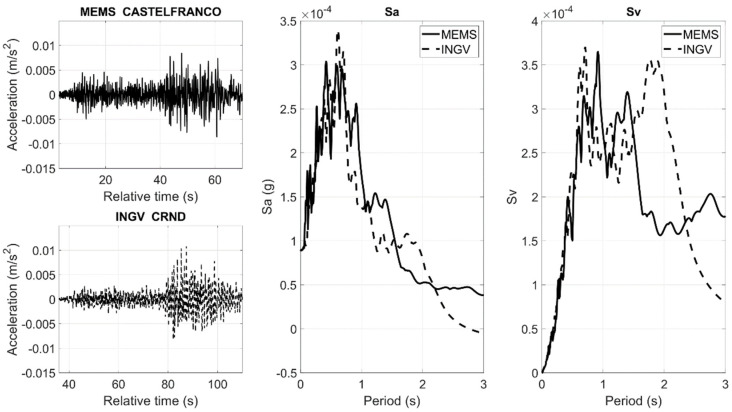
Comparison of the ASX1000 MEMS sensor prototype, CASTELFRANCO, and high-quality sensors, CRND, belonging to the INGV networks. The comparison is made in terms of waveforms, normalized Sa, and Sv.

Figure 6 and Figure 7 show the comparisons in terms of waveforms and 5% damped Sa and Sv, considering the X horizontal component. In many cases, the PGA values are comparable, as well as the shapes of Sa and Sv, as these parameters are strictly dependent on the local seismic effects.

## 4. Discussion

In this work, we evaluated the suitability of a new prototype of an inexpensive MEMS sensor, named ASX1000, to detect distant moderate seismic events. Research shows that these sensors are promising tools that could record local events [12] and small seismic events (with a magnitude in the order of 1.5 [18]). The experimental networks of MEMS accelerometers installed in northern Italy were also able to record a moderate seismic event (M_w_ = 6.3) that occurred 350 km away from the array, close to Petrinja in Croatia. The fundamental strong motion parameters (PGA, pseudo-absolute spectral velocity, and pseudo-absolute spectral acceleration) inferred from the accelerograms recorded by the MEMS sensors are comparable with the ones inferred from the closest high-quality seismic stations. The high-quality sensors considered here are conventional seismometers managed by the national institute INGV, which is in charge of national seismic monitoring.

The results show that the PGA values recorded are comparable, and the recorded signals are in reasonable agreement in the frequency domain. The normalized pseudo-spectral velocity and acceleration curves show similar trends, particularly for the shortest periods. ASX1000 responses are influenced by the higher self-noises of the MEMS sensors. Moreover, the national seismic network is deployed as insensitive to ambient noise sources (cultural and environmental), so that the sensitivity for the earthquake-generated signal is high. On the contrary, the ASX1000 prototypes are installed in urban contexts, within noisy telecommunication infrastructures. Nevertheless, they were still capable of recording such distant seismic events. The data show similarities in terms of ground motion parameters, and the compared sensors are located on similar site classes, even if the spatial variabilities in the positions of the ASX1000 prototypes and high-fidelity instruments are high. Moreover, due to the limited sensitivity and high internal noise of these prototypes, they cannot substitute high-quality conventional seismometers. In particular, these sensors seem to be poorly adaptable for typical seismological purposes (earthquake location, magnitude estimate, etc.). In the near future, we believe that new MEMS accelerometers could benefit from higher response performances (thanks to the quick industrial development of MEMS technology). Denser arrays at relatively low costs will significantly improve the construction of detailed shake maps, which is of relevant importance for early post-event management and seismic zonation studies. In this framework, there is an ongoing project regarding the installation of hundreds of new Adel MEMS sensors in the Veneto region, northern Italy (OGS-Regione del Veneto POR-FESR project). Considering the costs of the overall circuit components compared to the accelerometer ones, we are designing multi-sensor MEMS accelerometers to be used in a single station, to significantly improve the SNR of the acquired signals.

## Figures and Tables

**Table 1:** Technical specifications of the MEMS Adel prototype ASX1000.

Dynamic Range	±2 g
Resolution	3.9 μ
Non-linearity (range ±2 g)	0.1% over a total range of 4 g
Sensitivity (−40–125 °C)	±0.01%*/*°C
Noise density	25 μg/√Hz
Max RMS noise	0.197 mg
Max noise peak to peak	0.55 mg
Dimensions (mm)	Length 140, width 65, height 32
Power supply	36–60 V max, 160 mA max
Socket	N.8 simultaneous for LAN or Mobile Modem GPRS/3G/4G-LTE.
Storage	SD up to 64 Gbyte
Storage format	miniSeed, binary 24-bit, and csv

**Table 2:** PGA and PGV values recorded by the ASX1000 prototypes and the high-quality seismic sensors.

SENSOR—COMPONENT	PGA ASX1000 (m/s^2^)	PGV ASX1000 (m/s)	PGA HQ Sensors (m/s^2^)	PGV HQ Sensors (m/s)
CASTELFRANCO—CRND X	0.008	0.002	0.012	0.007
CASTELFRANCO—CRND Y	0.009	0.003	0.017	0.008
CASTELFRANCO—CRND Z	0.018	0.040	0.01	0.046
BASSANO—SANR X	0.009	0.0021	0.011	0.05
BASSANO—SANR Y	0.007	0.0017	0.05	0.063
BASSANO—SANR Z	0.015	0.034	0.09	0.081

## Data Availability

INGV (Istituto Nazionale di Geofisica e Vulcanologia) seismic catalog: (http://cnt.rm.ingv.it/event/25870121) (accessed on 29 March 2022); M6.4–2 km WSW of Petrinja, Croatia, ShakeMap. Available online: https://earthquake.usgs.gov/earthquakes/eventpage/us6000d3zh/shakemap/intensity (accessed on 15 March 2022).
